# Characterisation of burden of illness measures associated with human (Fluoro)quinolone-resistant *Campylobacter* spp. infections – a scoping review

**DOI:** 10.1017/S095026882200139X

**Published:** 2022-11-11

**Authors:** M. J. Isada, M. Reist, M. C. MacKinnon, F. C. Uhland, K. M. Young, K. Gibbens, E. J. Parmley, C. A. Carson

**Affiliations:** 1 Centre for Food-borne, Environmental and Zoonotic Infectious Diseases, Public Health Agency of Canada, Guelph, Ontario, Canada; 2 Veterinary Drugs Directorate, Health Products and Food Branch, Health Canada, Ottawa, Ontario, Canada; 3 Public Health Risk Sciences Division, National Microbiology Laboratory, Public Health Agency of Canada, Guelph, Ontario, Canada; 4 Toronto Western Hospital, University Health Network, Toronto, Ontario, Canada; 5 Department of Population Medicine, University of Guelph, Guelph, Ontario, Canada

**Keywords:** Burden of illness, *Campylobacter* spp., fluoroquinolone resistance, human infections, scoping review

## Abstract

*Campylobacter* spp. are one of the most common causes of bacterial gastroenteritis in Canada and worldwide. Fluoroquinolones are often used to treat complicated human campylobacteriosis and strains of *Campylobacter* spp. resistant to these drugs are emerging along the food chain. A scoping review was conducted to summarise how human (fluoro)quinolone-resistant (FQR; quinolones including fluoroquinolones) *Campylobacter* spp. infections are characterised in the literature by describing how burden of illness (BOI) associated with FQR is measured and reported, describing the variability in reporting of study characteristics, and providing a narrative review of literature that compare BOI measures of FQR *Campylobacter* spp. infections to those with susceptible infections. The review identified 26 studies that yielded many case reports, a lack of recent literature and a lack of Canadian data. Studies reported 26 different BOI measures and the most common were hospitalisation, diarrhoea, fever and duration of illness. There were mixed results as BOI measures reported in literature were inconsistently defined and there were limited comparisons between resistant and susceptible infections. This presents a challenge when attempting to assess the magnitude of the BOI due to FQR *Campylobacter* spp., highlighting the need for more research in this area.

## Introduction

*Campylobacter* spp. are one of the leading causes of foodborne illness with an estimated 447 cases per 100 000 persons annually in Canada, and 16% of foodborne illnesses globally [1, 2]. *Campylobacter* spp. infections are often self-limiting and are characterised by acute gastroenteritis, commonly diarrhoea, abdominal pain and fever, with severe illness leading to hospitalisation and septicaemia [3]. Common sources of *Campylobacter* spp. include contaminated food or water, primarily undercooked chicken [3].

Due to the self-limiting nature of campylobacteriosis, antimicrobial treatment is mainly recommended for cases with severe or prolonged infections and immunocompromised patients [4]. Fluoroquinolones are often used for treatment of complicated human *Campylobacter* spp. infections [5]. The term (fluoro)quinolone-resistant (FQR) used in this review refers to both the quinolones, a group of broad-spectrum bactericidal agents and fluoroquinolones, a class of quinolone-derived antibiotics, which include, but are not limited to ciprofloxacin, levofloxacin and moxifloxacin [6]. Fluoroquinolones are classified as ‘Category I: Very High Importance’ in human medicine by Health Canada's Veterinary Drugs Directorate (VDD) [7]. This classification indicates that fluoroquinolones are one of the limited available therapies for treating serious *Campylobacter* spp. infections which means resistance to these drugs may leave patients with few or no options for effective treatment [7]. Quinolones (except fluoroquinolones) are classified as ‘Category II: High Importance’ in human medicine by Health Canada's VDD [7]. Quinolones (including fluoroquinlones) are classified as ‘Critically Important Antimicrobials’ used in human medicine by the World Health Organization (WHO) [8]. The WHO listed ‘*Campylobacter*, fluoroquinolone-resistant’ as ‘High’ priority in the ‘WHO Priority Pathogens List for Research and Development of New Antibiotics’ [9].

Antimicrobial resistance (AMR) in bacteria, including *Campylobacter* spp., can develop from a genetic mutation within the organism or acquiring existing resistance genes from other organisms due to selection pressures from antimicrobial use [10]. Studies in Europe and the United States indicate that fluoroquinolone use in animals can select for resistance in human pathogens, particularly *Campylobacter jejuni*, and the incidence of human FQR *Campylobacter* spp. infections increased after these drugs were licensed for food animals use [11]. In 2005, the FDA removed approval for fluoroquinolone use in water for poultry based on a risk assessment that found the development of FQR *Campylobacter* spp. in poultry and its ability to be transferred to people [12]. A study found similar AMR profiles of *Campylobacter* spp. isolates from broiler chickens in Canadian farms with chicken meat retailed in different regions of Canada [13]. In Canada, fluoroquinolones are approved for treating respiratory disease in cattle and pigs, but are not approved for use in poultry [14]. With people, animals and the environment serving as reservoirs for the transmission of such resistance genes, infection with FQR *Campylobacter* spp. is known to spread through the food chain ultimately posing a threat to food safety and human health [11, 14, 15].

*Campylobacter jejuni* is the most common type of campylobacter bacteria associated with human campylobacteriosis, and other species reported in Canada include *C. coli*, *C. fetus*, *C. lari* and *C. upsaliensis* [16]. In a region of Western Canada, a study found that most infections were linked to *C. jejuni* subtypes associated with cattle, and many occurrences of pathogenic campylobacter species were not detected by the conventional laboratory methodology [17]. An additional study looking at campylobacteriosis in this region found that most FQR *C. jejuni* subtypes were endemic in Canada and mostly linked to cattle and chicken reservoirs [18]. These studies highlight the need to examine campylobacter at the species level to gain further understanding of reservoirs and transmission mechanisms. The utility of techniques in molecular epidemiology, including whole genome sequencing, will evolve research and enhance our ability to discriminate between strains and advance our assessment of impacts to health [17, 18].

Increases in FQR *Campylobacter* spp. have been observed in Canada and worldwide [10, 18, 19]. An increase in human FQR *Campylobacter* spp. isolates was observed in Canada between 2011 and 2018 [19]. This is concerning as the Council of Canadian Academies Expert Panel on the Potential Socio-Economic Impacts of AMR in Canada found that in 2018 AMR cost the Canadian healthcare system an estimated $1.4 billion [20]. Moreover, based on estimates, the Panel reported that resistant infections have a higher mortality rate compared to susceptible infections, likely due to complications from delays in obtaining effective treatment [20]. Burden of illness (BOI) is defined broadly as the direct and indirect impacts or costs of illness which can have clinical outcome and resource implications from the patient, healthcare system and economic perspective [21, 22]. To estimate the overall human health impact of FQR *Campylobacter* spp. infections, studies utilise a variety of BOI measures including duration of illness, treatment failure, absenteeism and direct/indirect healthcare costs [11, 23, 24].

A scoping review was undertaken to synthesise available literature. Scoping reviews are a key tool to summarise research findings and knowledge gaps to inform knowledge users, including policy makers and practitioners [25]. Currently, there is no publication that has synthesised BOI measures related to human FQR *Campylobacter* spp. infections that the authors are aware of. This synthesis of information will add to existing knowledge and subsequently aid in the development of risk assessments and risk reduction measures related to human FQR *Campylobacter* spp. infections. The purpose of this review is to summarise how FQR *Campylobacter* spp. infections are characterised in the literature by (i) describing how BOI associated with FQR is measured and reported; (ii) describing the variability in reporting of study characteristics; and (iii) providing a narrative synthesis of literature that compared BOI measures between FQR *Campylobacter* spp. infections and those with susceptible infections.

## Methods

### Literature search

This scoping review was guided by methodology from ‘Preferred Reporting Items for Systematic review and Meta-Analyses extension for Scoping Reviews’ (PRISMA-ScR) [26]. A protocol was established prior to starting the scoping review and updated prior to the second search (available upon request). A literature search was conducted to capture published BOI data on human infection with FQR *Campylobacter* spp. The terms included in the search string related to the bacteria of interest (campylobacte*r*), and the exposure terms ((fluoro)quinolone resistance), which were compiled by the research team using the WHO ATC/DDD Index (Table 1) [27]. Terms related to specific campylobacter species were not included to capture all possible camplylobacter infections regardless if they were speciated or if the species was not mentioned. Terms related to the outcome (BOI) were not included to capture all possible BOI measures. Seven databases were searched on 6 February 2020: MEDLINE® via Ovid, EMBASE® via Ovid, Core Collection in Web of Science, Scopus, CAB Abstracts via Ovid, AGRICOLA™ via Ovid and Global Health via Ovid. Grey literature sources included the WHO's Global Index Medicus and Google Scholar (only first 250 results based on relevance). The primary and grey literature searches were repeated on 7 June 2021 and 10 June 2021, respectively (Table S1), both with a publication date limit of 2020 to present. In the updated search, MEDLINE® via Ovid and EMBASE® via Ovid were run without the human filter and with a publication date filter from 2018-present. This was done to ensure that all articles were captured even if there was a delay in indexing (time interval between entry into database and indexing) [28].

**Table 1.** Search string of for primary search of Scopus (February 2020) used to identify literature associated with (fluoro)quinolone-resistant *Campylobacter* spp. infections

#	Searches: Scopus (6 February 2020)	Results	Annotations
1	TITLE-ABS-KEY (campylobacter*)	25 055	Bacteria of interest – campylobacter terms
2	TITLE-ABS-KEY (‘drug resistance’ OR resistan*)	2 383 554	Exposure – Resistance terms
3	TITLE-ABS-KEY (fluoroquinolone$ or quinolone$ or besifloxacin$ or cinoxacin$ or ciprofloxacin$ or danofloxacin$ or delafloxacin$ or difloxacin$ or enoxacin$ or enrofloxacin$ or fleroxacin$ or flumequin$ or garenoxacin$ or gatifloxacin$ or gemifloxacin$ or grepafloxacin$ or ibafloxacin$ or levofloxacin$ or lomefloxacin$ or moxifloxacin$ or nadifloxacin$ or ‘nalidixic acid’ or nemonoxacin$ or norfloxacin$ or ofloxacin$ or orbifloxacin$ or ozenoxacin$ or ‘oxolinic acid’ or pazufloxacin$ or pefloxacin$ or ‘pipemidic acid’ or ‘piromidic acid’ or pradofloxacin$ or prulifloxacin$ or rosoxacin$ or rufloxacin$ or sitafloxacin$ or sparfloxacin$ or temafloxacin$ or tosufloxacin$ or trovafloxacin$)	187 570	Exposure – (Fluoro)quinolone terms
4	2 and 3	76 023	Exposure – FQR
5	1 and 4	1969	Outcome – FQR campylobacter

### Inclusion criteria

Articles in this review were included based on the following criteria: population, exposure/comparator, outcome, study design and language. To be included, studies needed to be about people of any age with a laboratory confirmed infection with any FQR-*Campylobacter* spp. The comparator group, when appropriate to the study design, included cases with *Campylobacter* spp. infections that were either susceptible to (fluoro)quinolones or pan-susceptible. The studies must have also evaluated one or more BOI measures that included, but were not limited to, treatment failure, mortality and length of hospital stay (LOS). Lastly, the included studies had to be primary observational studies [29], including theses and dissertation, and published in English or French.

### Relevance screening

All citations were imported and de-duplicated in RefWorks© (ProQuest, Ann Arbor, United States) and DistillerSR® (Evidence Partners, Ottawa, Canada). After removal of duplicates, primary screening of titles and abstracts was performed using the inclusion criteria (Table S2). An accelerated screening approach was utilised where articles included by a single reviewer proceeded to secondary screening and agreement by two reviewers was required for excluded references. Secondary screening of full-text was completed by two reviewers independently with inclusion criteria reapplied (Table S3). Conflicts were discussed and resolved between reviewers and if necessary, arbitrated by a third researcher with inclusion criteria applied.

### Data extraction

Data extraction was performed by two reviewers individually in DistillerSR®, and conflicts were managed the same as during screening. Descriptive data that were extracted included study characteristics (year of publication, year(s) of data collection, study design, country, study objective, study site(s), campylobacter species), study participant characteristics (underlying common disease process and/or common characteristics, immune status, definition of resistant infections, numbers and selection of cases and comparators, age and sex, type of infections, testing methods and criteria) and BOI measures reported (definitions and results) (Table S4). BOI measures were mapped and qualitatively assessed according to three perspectives outlined in a systematic review by Naylor *et al*. [21]. These perspectives included patient perspective (e.g., mortality and morbidity measures), healthcare system perspective, which looks at the burden to healthcare providers (e.g. LOS and hospital costs), and economic perspective, which looks at the impact on the labour force and society (e.g., absenteeism) [21]. All of these data were synthesised to provide a qualitative summary of BOI measures in the literature that related to human FQR *Campylobacter* spp. infections.

## Results

### General

**Fig. 1.**
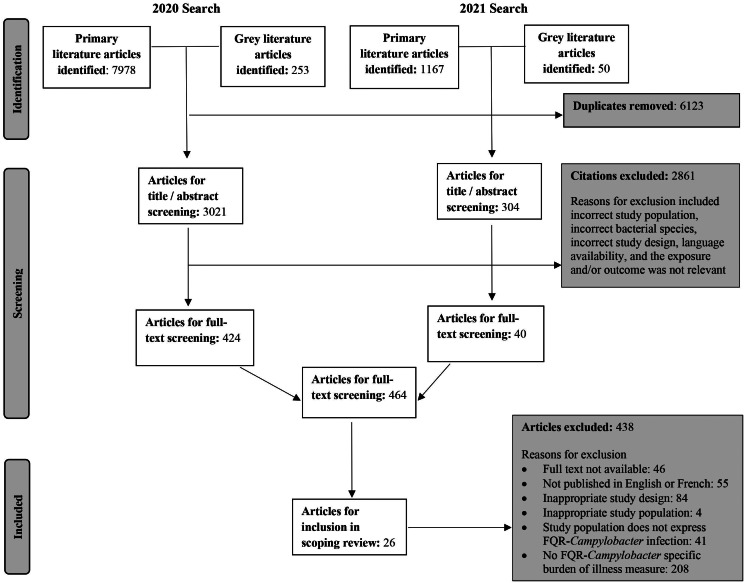
Literature search and screening process for a scoping review of BOI measures related to human (fluoro)quinolone-resistant *Campylobacter* spp. infections (adapted from [26]).

**Fig. 2.**
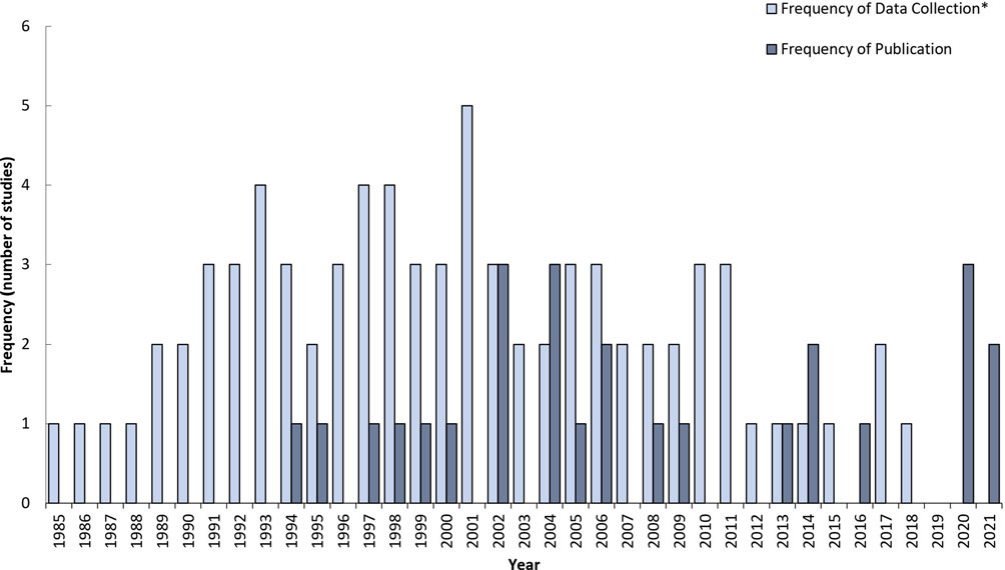
Years of data collection and publication period for data included in the scoping review of BOI measures related to human (fluoro)quinolone-resistant *Campylobacter* spp. infections. *Frequency of Data Collection: each year of data during a multi-year collection period is recorded as one value.

The original and updated literature searches retrieved 3325 (de-duplicated) potentially relevant articles, out of these 26 articles met inclusion criteria and were identified for data extraction (Fig. 1). The data from included articles were collected from 1985 to 2018 and published from 1994 to 2021 (Fig. 2). The most common author reported study designs were case reports (*n* = 11) and case-comparisons (*n* = 4). The studies were conducted in 15 different countries with the United States (*n* = 5) and Japan (*n* = 3) as the most common (Tables 2 and 3). In terms of *Campylobacter* spp, the most commonly reported were *C. jejuni* (*n* = 16) and *Campylobacter coli* (*n* = 7), and six studies did not report the specific species (Tables 2 and 3). Non-case reports (*n* = 15) were mostly conducted in multi-sites (*n* = 9) and all case reports were conducted in a single site (Tables 2 and 3). Most studies reported underlying common disease process and/or common characteristics (*n* = 19), including various comorbidities such as cancer (*n* = 5), human immunodefiency virus (HIV) (*n* = 2) and diabetes (*n* = 2) (Tables 2 and 3). The most common types of infection reported were gastrointestinal (GI) (*n* = 22) and blood-stream infection (BSI) (*n* = 8) (Tables 2 and 3). The number of cases with resistant infections ranged from 1 to 1078 and the number of cases with susceptible infections ranged from 3 to 3726 (Table 2). Cases and comparators were identified using various methods, including hospital medical records, patient registries, institutional databases and questionnaire responses.

**Table 2.** Characteristics of references (excluding case reports) included in scoping review of BOI measures related to human (fluoro)quinolone-resistant *Campylobacter* spp. infections

Reference	Author reported study design	Type and no. of sites	Country of study	Campylobacter species	Underlying disease process and/or common characteristics	Type of infection	No. of cases with resistant infections	No. of cases with susceptible infections	How were cases and comparators selected?
*Campylobacter* Sentinel Surveillance Scheme Collaborators [36]	Case-case analysis	Multi-site (≥3)	England and wales	*C. jejuni*	Explored: −Travel associated −Indigenous cases	GI	Travel: 347/495 Indigenous: 291/1884	Travel: 148/495 Indigenous: 1593/1884	No selection
Engberg *et al*. [35]	Prospective study	Multi-site (≥3)	Denmark (Copenhagen and Funen county)	*C. coli, C. jejuni*	NR	Non-specific infections	127/672	545/672	All culture-positive *Campylobacter* spp. infections were included, but linked microbiological and epidemiologic data were available for 69.5% of patients.
Evans *et al*. [30]	Case-comparison	Multi-site (≥3)	United Kingdom (Wales)	*Campylobacter* spp.	Explored: −Diabetes −Pre-existing bowel disease −Foreign travel in 7 days before onset	GI	145/556	411/556	All patients with CIP-R *Campylobacter* spp. isolated from faeces enroled and comparators with CIP-S *Campylobacter* spp. isolated randomly selected (unmatched study).
Feodoroff *et al*. [38]	Case series	Single-site	Finland	*C. coli, C. jejuni*	Explored impact of having or not having underlying disease on being treated at hospital for at least 2 days, and having severe symptoms	GI	97/192	95/192	Each patient positive for *C. jejuni* or *C. coli* received a questionnaire. Sample size for each data item was related to responses received.
Gaudreau *et al*. [52]	Retrospective case study	Single-site	Canada	*C. coli*	−All age 20–59 −All male Explored: −HIV positive −Sexual relations within incubation period −MSM	GI	10/10	N/A	All cases infected with an erythromycin-susceptible, tetracycline- and ciprofloxacin-resistant *C. coli* pulsovar 1 were enroled.
Gupta *et al*. [37]	Retrospective case-comparison study	Multi-site (≥3)	United States	*Campylobacter* species unspecified	NR	GI	16/47	31/47	All CIP-R *Campylobacter* spp. infections identified were enroled and matched with up to two CIP-S *Campylobacter* spp. infections by geographic site and date of stool specimen collection.
Helms *et al*. [41]	Cohort	Multi-site (≥3)	Denmark	*Campylobacter* spp.	NR	Non-specific infections	856/3471 (96 also erythromycin resistant)	2615/3471	No selection – cohort from registry of *Campylobacter* spp. infection.
Molina *et al*. [40]	Retrospective/Prospective case analysis	Single-site	France	*C. coli, C. jejuni*	−All had AIDS −All chronic *Campylobacter* infection	GI	1/4	3/4	No selection-cohort of HIV infected patients with *Campylobacter* infections.
Mori *et al*. [53]	NR	Single-site	Japan	*C. jejuni*	−5 had underlying diseases	GI, BSI	3/6	3/6	No selection – all cases of blood culture-confirmed *C. jejuni* bacteremia were enroled.
Nelson *et al*. [33]	Case-comparison	Multi-site (≥3)	United States	*Campylobacter* spp.	−29 (4%) had preexisting medical conditions	GI	82/740	658/740	Included persons who had been interviewed for a FoodNet case-control study and had an isolate tested for susceptibility. No selection for case-comparison study – included all persons with CIP-R and CIP-S *Campylobacter* spp.
Ricotta *et al*. [47]	NR	Multi-site (≥3)	United States	*Campylobacter* spp.	−*Campylobacter* spp. in persons with international travel in week prior to illness compared with cases in individuals with no international travel	Non-specific infections	1078/4804	3726/4804	No selection – Included all campylobacteriosis cases with known travel status and NARMS AMR data available.
Sanders *et al*. [39]	Cross-sectional	Two-sites	Thailand	*C. coli, C. jejuni*	−Most (92%) were male	GI	22/23	1/23	Identified patients with CIP-R and CIP-S *Campylobacter* spp. isolated from stool.
Smith *et al*. [34]	Case-comparison	Single-site	United States	*C. jejuni*	NR	GI	130/390	260/390	Each patient with a resistant isolate was matched with 2 patients with sensitive isolates; matched for age, residence and date of specimen collection.
Tee & Mijch [31]	NR	Single-site	Australia	*C. jejuni*	Explored: -if HIV-infected All HIV positive patients were homosexual or bisexual men.	GI, BSI	HIV: 2/9 Non-HIV: 1/12	HIV: 7/9 Non-HIV: 11/12	Reviewed records and identified 24 cases of culture-confirmed *C. jejuni* bacteremia, 21 had sufficiently detailed medical records and were included.
Unicomb *et al*. [32]	Period-prevalence survey	Multi-site (≥3)	Australia	*C. jejuni*	NR	GI	Locally acquired: 14/574 Overseas acquired: 7/11 Total: 21/585	Locally acquired: 560/574 Overseas acquired: 4/11 Total: 564/585	*Campylobacter* spp. isolates were identified from laboratory reports from 5 Australian states (states require doctors and labs to report patients infected with *Campylobacter* spp.). Isolates were tested for susceptibility to ciprofloxacin.

BSI, Bloodstream infection; CIP-R, Ciprofloxacin-resistant; CIP-S, Ciprofloxacin-susceptible; GI, Gastrointestinal; HIV, Human immunodeficiency virus; MSM, Men sexually active with men; NARMS, National Antimicrobial Resistance Monitoring System for Enteric Bacteria; NR, Not reported; N/A. Not available.

**Table 3.** Characteristics of case reports included in scoping review of BOI measures related to human (fluoro)quinolone-resistant *Campylobacter* spp. infections

Reference	Country of study	Campylobacter species	Underlying disease process and/or common characteristics	Type of Infection	Age and Sex
Dan & Parizade [54]	Israel	*C. coli*	X-linked agammaglobulinemia in childhood	GI	29 Year old male
Goyal *et al*. [55]	United States	*C. jejuni*	Patient 1: coronary artery disease, type 2 diabetes mellitus, hypertension, Patient 2: pancreatic cancer, pancreaticoduodenectomy and cholecystectomy	GI	67 Year old male, 60 Year old female
Hartman *et al*. [43]	Netherlands	*C. jejuni*	Lymphoplasmacytic lymphoma	GI, BSI, bilateral femoral osteomyelitis	54 Year old female
Kaneko *et al*. [56]	Japan	*C. jejuni*	NR	GI, BSI	14 Year old male
Kotilainen *et al*. [57]	Finland	*Campylobacter spp.*	NR	GI, myopericarditis	47 Year old male
Lau *et al*. [58]	Hong Kong (China)	*C. jejuni*	Two cases of bone marrow transplantation during post-graft period; Case 1: chronic myeloid leukaemia, Case 2: stage IV rhabdomyosarcoma	GI, BSI	15 Year old male, 17 Month old male
Magaz-Martinez *et al*. [44]	Spain	*C. jejuni*	Acute renal failure	GI	78 Year old male
Martora *et al*. [42]	Italy	*C. jejuni*	Acute lymphoblastic leukaemia	GI, BSI	4 Year old female, 7 Year old male
Nishikubo *et al*. [59]	Japan	*C. fetus*	Chronic atrial fibrillation, hypertension and right putamen haemorrhage without apparent sequelae	BSI, breast implant infection	64 Year old female
Pascual *et al*. [46]	Spain	*C. coli*	Three episodes of UTI since 3 months old	GI, UTI	9 Month old female
Tajada *et al*. [45]	Spain	*C. jejuni*	Immunocompetent child undergone a tonic-clonic generalised seizure within 12 h of onset of a febrile syndrome and pharyngotonsillitis	GI, BSI	1 Year old female

BSI, Bloodstream infection; GI, Gastrointestinal; UTI, Urinary tract infection.

Case reports detailed the age and sex of participants, where age ranged from 9 months to 78 years and they included six females and eight males (Table 3). Age and sex characteristics were reported by 13 non-case report studies (Table S5). Studies defined cases with FQR infections as being resistant to fluoroquinolones (*n* = 17), quinolones (*n* = 4) or both (*n* = 5) (Table S6). They described the antimicrobial susceptibility testing method, where the most common method was disk diffusion (*n* = 8), and the most common interpretive criteria used were the Clinical and Laboratory Standards Institute clinical breakpoints (*n* = 8) (Table S6). Studies reported 26 different BOI measures, which related to the BOI perspective of the patient (*n* = 23), healthcare system (*n* = 2) or economy (*n* = 1). The most commonly identified BOI measures across all studies were hospitalisation, diarrhoea, fever and duration of illness (Table 4).

**Table 4.** Identified BOI measures for human (fluoro)quinolone-resistant *Campylobacter* spp. infections (*n* = 26 references)

BOI Perspective	BOI measures category	Number of references (excluding case reports)	Number of case reports	Total number of references
Patient	Diarrhoea	8	9	17
	Fever	7	8	15
	Duration of illness	8	4	12
	Bloody stool	6	2	8
	Bacteremia	2	6	8
	Abdominal pain	3	3	6
	Treatment failure	1	4	5
	Vomiting	4	0	4
	Complications	0	4	4
	Mortality	2	1	3
	Nausea	1	2	3
	Other:			
	Muscle aches	1	0	1
	Painful joints	1	0	1
	Weakness	1	0	1
	Headache	1	0	1
	Adverse events (invasive illness or death)	1	0	1
	Ileocolitis	0	1	1
	Pharyngotonsillitis	0	1	1
	Urinary tract infection	0	1	1
	Progressive pain in upper extremities due to osteomyelitis	0	1	1
	Reduced appetite	0	1	1
	Night sweats	0	1	1
	Severe polar	0	1	1
Healthcare system	Hospitalisation	11	9	20
	Length of hospital stay	3	4	7
Economic	Absenteeism	2	0	2

### Patient perspective BOI measures

#### Diarrhoea

Diarrhoea was the most commonly reported patient perspective BOI measure (*n* = 17). There were several definitions of diarrhoea used including having three or more loose stools in a 24 h period (*n* = 1), two or more loose stools in a 24 h period with one or more associated GI symptom (*n* = 1), chronic diarrhoea (three or more bowel movements per day lasting more than two days) (*n* = 1) and frequency of diarrhoea (*n* = 1). The remaining 13 studies did not provide a definition. Two studies presented comparisons between FQR and susceptible cases. One study found no difference between the two groups [30], and one study reported diarrhoea in HIV positive and HIV negative patients for both FQR and susceptible cases [31] (Table S7). The remaining studies did not provide a comparison between FQR and susceptible infections and only mentioned participants reporting diarrhoea (*n* = 12), or all participants enroled in the study had diarrhoea (*n* = 2).

#### Fever

Fever was the second most commonly reported patient perspective BOI measure (*n* = 15). A fever was defined as having a temperature of ≥38 °C (*n* = 4), or no definition was provided (*n* = 11). Four studies presented comparisons between FQR and susceptible cases. One study found that FQR cases were less likely to report fever than cases with susceptible infections [30] (Table S7). One study reported fever in HIV positive and HIV negative patients for both FQR and susceptible cases [31], and two studies found no difference in fever between the FQR and susceptible cases [32, 33]. The remaining studies did not provide a comparison between FQR and susceptible infections and only mentioned participants reporting a fever (*n* = 11).

#### Duration of illness

Duration of illness was the third most commonly reported patient perspective BOI measure (*n* = 12). Duration of illness was defined as duration of diarrhoea in days and/or months (*n* = 7), duration of diarrhoea and fever in days (*n* = 1), duration of diarrhoea and chest pain in days (*n* = 1), or no definition was provided (*n* = 3). Eight studies presented comparisons between FQR and susceptible cases. Three studies found a longer duration of illness in resistant cases compared to susceptible cases, and two of the results were statistically significant [33–35] (Table S7). One study reported duration of diarrhoea and fever in HIV positive and HIV negative patients for both FQR and susceptible cases [31], and four studies found no difference in duration of illness between the FQR and susceptible cases [30, 32, 35, 36] (Table S7). The remaining studies did not provide a comparison between FQR and susceptible infections and only mentioned participants reporting duration of illness (*n* = 5).

#### Bloody stool

Bloody stool was a reported patient perspective BOI measure by eight studies. Bloody stool was defined as blood found in a stool specimen (*n* = 1), bloody diarrhoea (*n* = 1), or no definition was provided (*n* = 6). Five studies presented comparisons between FQR and susceptible cases. One study reported a higher percentage of FQR cases having bloody diarrhoea compared with susceptible cases [37] (Table S7). In contrast, one study found that susceptible isolates seemed to cause bloody stools more than FQR isolates [38] (Table S7). Three studies found no difference between FQR and susceptible cases [30, 32, 33] (Table S7). The remaining studies only mentioned participants reporting bloody stool (*n* = 3).

#### Bacteremia

Bacteremia or BSI was a reported patient perspective BOI measure by eight studies. Bacteremia was defined as a positive blood culture for *Campylobacter* spp. (*n* = 7), or no definition was provided (*n* = 1). One study presented bacteremia in both FQR and susceptible cases, however due to the study design all participants had bacteremia [31]. The remaining studies only mentioned participants reporting bacteremia irrespective of susceptibility (*n* = 7).

#### Abdominal pain

Abdominal pain was a reported patient perspective BOI measure by six studies. There were no definitions provided. Two studies presented comparisons between FQR and susceptible cases and both studies found no difference between the two groups [30, 33] (Table S7). The remaining studies only mentioned participants who reported abdominal pain irrespective of susceptibility (n = 4).

#### Treatment failure

Treatment failure was a reported patient perspective BOI measure by five studies (Table S7). Treatment failure was defined as lack of resolution of diarrhoea with standard antibiotic treatment (*n* = 1), persistence of diarrhoea, bacteremia and femoral pain (*n* = 1), or no definition was provided (*n* = 3). One study compared treatment failure between FQR and susceptible infections and found higher treatment failure among the FQR cases [39] (Table S7). The remaining studies only described treatment failure irrespective of susceptibility (*n* = 4).

#### Vomiting

Vomiting was a reported patient perspective BOI measure by four studies. There were no definitions provided. Three studies presented comparisons between FQR and susceptible cases and all found no difference between the two groups [30, 32, 33] (Table S7). The remaining study only mentioned participants who reported vomiting.

#### Complications

Complications were a reported patient perspective BOI measure by four studies (Table S7). There were no definitions provided and no comparisons between FQR and susceptible infections were made.

#### Mortality

Mortality was a reported patient perspective BOI measure by three studies. Mortality was defined as death (*n* = 2), or death during follow-up (*n* = 1). Two studies compared mortality between FQR and susceptible cases, where one study reported cases of death in HIV positive and HIV negative patients for both FQR and susceptible cases [31], and the other study reported the number of deaths in both FQR-resistant and susceptible groups [40] (Table S7). The remaining study did not provide a comparison between FQR and susceptible infections.

#### Nausea

Nausea was a reported patient perspective BOI measure by three studies. There were no definitions provided. One study presented comparisons between FQR and susceptible cases and found no difference between the two groups [30] (Table S7). The remaining studies only mentioned participants who reported nausea irrespective of susceptibility (*n* = 2).

#### Other

There were additional BOI measures under the patient perspective category documented by one included study (Table 4). Two studies presented comparisons between FQR and susceptible cases. One study found that patients with FQR infection had an increased risk of adverse events (a diagnosis of either invasive illness or death) compared with patients with susceptible strains [41] (Table S7). One study reported comparisons between FQR and susceptible cases with regard to muscle aches, painful joints, weakness and headache, but found no significant difference between these groups for any of these outcomes [30] (Table S7). The remaining BOI measures, reduced appetite, night sweats, severe polar, progressive pain in upper extremities due to osteomyelitis, ileocolitis, pharyngotonsillitis and urinary tract infection, were observed in five case reports [42–46] (Table S7).

### Healthcare system perspective BOI measures

#### Hospitalisation

Hospitalisation was the most commonly reported healthcare system perspective BOI measure (*n* = 20). Hospitalisation was defined as admission to the hospital (*n* = 11), requiring hospitalisation (*n* = 2), presence of a hospital medical record (*n* = 1) or no definition was provided (*n* = 6). Seven studies presented comparisons between FQR and susceptible cases. Two studies reported that patients with FQR infections were more likely to be hospitalised compared to patients with susceptible infections, where one study looked at non-travel cases [37, 47] (Table S7). Two studies found that hospitalisation was higher with susceptible cases compared to FQR cases, where one study looked at international travel cases [38, 47] (Table S7). Four studies found no difference between FQR and susceptible infections [30, 32, 33, 36] (Table S7). The remaining studies only mentioned participants being hospitalised irrespective of susceptibility (*n* = 13).

#### Length of hospital stay

LOS was the only other reported healthcare system perspective BOI measure (*n* = 7). It was defined as LOS in days (*n* = 2), days from admission to discharge (*n* = 2), time from admission to death (*n* = 1) or no definition was provided (*n* = 2). Two studies presented comparisons between FQR and susceptible cases. One study found that the mean LOS was longer for the susceptible group than for the FQR group [33] (Table S7). However, one study did not find a difference between median LOS in FQR and susceptible cases [32] (Table S7). The remaining studies only mentioned LOS irrespective of susceptibility (*n* = 4).

### Economic perspective BOI measures

#### Absenteeism

Absenteeism was the only reported economic perspective BOI measure (*n* = 2). Absenteeism was defined as missed or time off work or school (*n* = 1) and missed usual activities in days (*n* = 1). Both of the studies presented comparisons between FQR and susceptible cases and found no difference between both groups [30, 33] (Table S7).

## Discussion

This scoping review summarised literature that reported BOI measures associated with human FQR *Campylobacter* spp. infections three categories (patient, healthcare system and economic perspectives [21]). The majority of studies reported patient perspective BOI measures, and we found a limited amount of information in the healthcare system and economic BOI perspectives. There were no studies that addressed healthcare costs, which would be useful to gain further insight since there were many hospitalisations associated with FQR *Campylobacter* spp. infections.

While our scoping review was not specifically designed to evaluate comorbidities, several studies provided insight into BOI due to FQR *Campylobacter* spp. infection in patients with underlying health conditions, and the most commonly reported were cancer, HIV and diabetes. This knowledge can be important in the clinical care of patients, and may also provide an opportunity for future research into identification and exploration of potential high risk populations in relation to patients with FQR *Campylobacter* spp. infections.

Similar to *Campylobacter* spp., there has been research for which BOI related to resistance has been explored in other enteric pathogens. *Salmonella* spp. are another common cause of foodborne illness that result in gastroenteritis in Canada and globally [48]. A Canadian study found an increased risk of hospitalisation for cases with multidrug-resistant *Salmonella enterica* serotype Typhimurium compared to cases with susceptible infections [48]. In a Danish study, patients infected with quinolone-resistant *S. enterica* serotype Typhimurium had a higher risk of invasive illness or death within 90 days of infection than patients infected with pansusceptible strains [49]. In the United States, a study found that BSI occurred more frequently among patients infected with drug resistant non-typhoidal *Salmonella* than patients with pan-susceptible infection [50]. We are not aware of a published manuscript synthesising BOI measures relating to AMR in non-typhoidal *Salmonella* and this could be an area for future research.

An important question that clinicians, risk assessors and policy makers seek answers to is whether BOI measures increase with FQR *Campylobacter* infections when compared to those with susceptible infections? Although answering this question is beyond the scope of our review, we can provide insight into the ability to answer this question with a future systematic review and meta-analysis. The most common study design was case reports and due to their design they did not provide data that compared resistant and susceptible cases. Even among the studies that were not case reports (*n* = 16), only some studies provided data that compared both groups (*n* = 11) and they all yielded mixed results. Duration of illness (*n* = 8) and hospitalisation (*n* = 7) had the most studies that provided data comparing resistant and susceptible cases. It is possible that a systematic review and meta-analysis could be performed on these two BOI outcomes, however, the heterogeneity of the studies would need to be assessed to evaluate whether it is appropriate to combine the results from the studies. As well, risk of bias would need to be assessed to provide the context necessary for interpreting the meta-analysis results. Our scoping review highlights the need for more well designed studies, which could inform future meta-analyses.

Another difficulty is the lack of standard reporting of BOI measures, as all studies employ different methodologies and reporting metrics. Study designs varied from case reports to large cohort studies where definitions of BOI measures varied and many studies did not provide definitions. These same obstacles have been discussed in recent reviews estimating the global health and economic burden of disease due to AMR in many pathogens [21, 51]. These studies state that in order to improve the precision of such estimates and in turn allocate resources and inform policy, broad scale changes in the collection and reporting of BOI measures may be necessary [21, 51].

In addition to the limitations addressed in previous paragraphs, other limitations of this review include the small number of studies included for synthesis, the lack of Canadian literature, and the lack of recent literature comparing resistant and susceptible groups. Only 26 publications met the inclusion criteria, identifying current knowledge gaps regarding human FQR *Campylobacter* spp. infections. Only one paper included was published in Canada and it reported BOI outcomes including abdominal cramps, bloody stool, diarrhoea, fever and hospitalisation [52]. However, this study did not compare between resistant and susceptible groups, thus more research is needed to understand BOI associated with FQR *Campylobacter* spp. in the Canadian context. There is a lack of recent literature comparing resistant and susceptible groups, with the most recent studies included that compared the two groups being published in 2009.

In conclusion, several types of BOI measures were reported in the literature to describe the human health impact of FQR *Campylobacter* spp. infections, however they were inconsistently defined. Despite the common occurrence of this foodborne pathogen, and particularly because of the limited amount of literature comparing BOI for FQR and susceptible *Campylobacter* spp. infections, this review illustrates the need for further research in which comparable or standardised measures of BOI are reported with harmonised approaches in study methodology. Key information that clinicians and researchers should collect to assess BOI from FQR *Campylobacter* spp. infections include details on the infection (e.g. sample source, species, method used for antimicrobial susceptibility testing (AST), AST interpretive criteria), patient characteristics including confounders (e.g. age, sex, co-morbidities/underlying disease), and clearly defined BOI measures (e.g. mortality, morbidity, healthcare system and economic impact) stratified by resistant and susceptible cases (Table S8). This information could lead to a better understanding of BOI due to human FQR *Campylobacter* spp. infections to inform knowledge users including clinicians, policy makers and risk assessors.

## Data Availability

The authors confirm that the data needed to replicate the findings of this study are available within its supplementary materials.

## Supplementary material

For supplementary material accompanying this paper visit http://doi.org/10.1017/S095026882200139X. click here to view supplementary material
