# Supplementary material for: Characterisation of burden of illness measures associated with human (Fluoro)quinolone-resistant Campylobacter spp. infections – a scoping review
Source: Epidemiol Infect. 2022 Nov 11;150:e205. doi: 10.1017/S095026882200139X (PMC9980926; doi:10.1017/S095026882200139X)
Supplement: Supplementary file 1 [file S095026882200139Xsup001.docx]

Epidemiology and Infection

**Characterization of burden of illness measures associated with human (Fluoro)quinolone-resistant *Campylobacter* spp. infections - A scoping review**

M. J. Isada^1*^, M. Reist^2^, M. C. MacKinnon^1^, F. C. Uhland^1^, K. M. Young^3^, K. Gibbens^4^, E. J. Parmley^5^, and C. A. Carson^1^

^1^Centre for Food-borne, Environmental and Zoonotic Infectious Diseases, Public Health Agency of Canada, Guelph, Ontario, Canada

^2^Veterinary Drugs Directorate, Health Products and Food Branch, Health Canada, Ottawa, Ontario, Canada

^3^Public Health Risk Sciences Division, National Microbiology Laboratory, Public Health Agency of Canada, Guelph, Ontario, Canada.

^4^Toronto Western Hospital, University Health Network, Toronto, Ontario, Canada

^5^Department of Population Medicine, University of Guelph, Guelph, Ontario, Canada

**Supplementary Material**

Table of Contents

[**Table S1**. Search strings in the updated search (June 2021) used to identify literature associated with human (fluoro)quinolone-resistant *Campylobacter* spp. infections 1](#_Toc101887309)

[**Table S2**. Primary screening form at the title/abstract level to identify literature associated with human (fluoro)quinolone-resistant *Campylobacter* spp. infections 4](#_Toc101887310)

[**Table S3.** Secondary screening form at the full-text level to identify literature associated with human (fluoro)quinolone-resistant *Campylobacter* spp. infections 5](#_Toc101887311)

[**Table S4.** Data extraction form annotations and an a-priori record of decisions to identify literature associated with human (fluoro)quinolone-resistant *Campylobacter* spp. infections 5](#_Toc101887312)

[**Table S5**. Age and sex characteristics of references (excluding case reports) included in scoping review of BOI measures associated with human (fluoro)quinolone-resistant *Campylobacter* spp. infections 8](#_Toc101887313)

[**Table S6**. Definitions of (fluoro)quinolone resistant infections, method used for antimicrobial susceptibility testing (AST), and AST interpretive criteria identified in all references included in scoping review of BOI measures associated with human (fluoro)quinolone-resistant *Campylobacter* spp. infections 10](#_Toc101887314)

[**Table S7**. Identified comparisons of BOI measures and other findings in human (fluoro)quinolone-resistant and susceptible *Campylobacter* spp. infections 12](#_Toc101887315)

[**Table S8**. Key information to collect when assessing BOI from (fluoro)quinolone-resistant and susceptible *Campylobacter* spp. infections in humans 18](#_Toc101887315)

# **Table S1**. Search strings in the updated search (June 2021) used to identify literature associated with human (fluoro)quinolone-resistant *Campylobacter* spp. infections

| **Database** | **Search Date*** | **Search String** |
| --- | --- | --- |
| MEDLINE® via Ovid | June 7^th^, 2021 | 1. exp campylobacter/ or campylobacter$.ab,kf,ti. 2. exp drug resistance, microbial/ or resistan$.ab,kf,ti. 3. quinolones.sh,xs. or (fluoroquinolone$ or quinolone$ or besifloxacin$ or cinoxacin$ or ciprofloxacin$ or danofloxacin$ or delafloxacin$ or difloxacin$ or enoxacin$ or enrofloxacin$ or fleroxacin$ or flumequin$ or garenoxacin$ or gatifloxacin$ or gemifloxacin$ or grepafloxacin$ or ibafloxacin$ or levofloxacin$ or lomefloxacin$ or moxifloxacin$ or nadifloxacin$ or nalidixic acid or nemonoxacin$ or norfloxacin$ or ofloxacin$ or orbifloxacin$ or ozenoxacin$ or oxolinic acid or pazufloxacin$ or pefloxacin$ or pipemidic acid or piromidic acid or pradofloxacin$ or prulifloxacin$ or rosoxacin$ or rufloxacin$ or sitafloxacin$ or sparfloxacin$ or temafloxacin$ or tosufloxacin$ or trovafloxacin$).ab,kf,ti. 4. 2 and 3 5. 1 and 4 6. limit 5 to yr="2018 -Current" |
| EMBASE® via Ovid | June 7^th^, 2021 | 1. exp campylobacter/ or campylobacter$.ab,kw,ti. 2. exp antibiotic resistance/ or resistan$.ab,kw,ti. 3. Exp quinolone antiinfective agent/ or (fluoroquinolone$ or quinolone$ or besifloxacin$ or cinoxacin$ or ciprofloxacin$ or danofloxacin$ or delafloxacin$ or difloxacin$ or enoxacin$ or enrofloxacin$ or fleroxacin$ or flumequin$ or garenoxacin$ or gatifloxacin$ or gemifloxacin$ or grepafloxacin$ or ibafloxacin$ or levofloxacin$ or lomefloxacin$ or moxifloxacin$ or nadifloxacin$ or nalidixic acid or nemonoxacin$ or norfloxacin$ or ofloxacin$ or orbifloxacin$ or ozenoxacin$ or oxolinic acid or pazufloxacin$ or pefloxacin$ or pipemidic acid or piromidic acid or pradofloxacin$ or prulifloxacin$ or rosoxacin$ or rufloxacin$ or sitafloxacin$ or sparfloxacin$ or temafloxacin$ or tosufloxacin$ or trovafloxacin$).ab,kw,ti. 4. 2 and 3 5. 1 and 4 6. limit 5 to yr="2018 -Current" |
| Scopus | June 7^th^, 2021 | 1. TITLE-ABS-KEY ( campylobacter*) 2. TITLE-ABS-KEY (“drug resistance” OR resistan*) 3. TITLE-ABS-KEY (fluoroquinolone$ or quinolone$ or besifloxacin$ or cinoxacin$ or ciprofloxacin$ or danofloxacin$ or delafloxacin$ or difloxacin$ or enoxacin$ or enrofloxacin$ or fleroxacin$ or flumequin$ or garenoxacin$ or gatifloxacin$ or gemifloxacin$ or grepafloxacin$ or ibafloxacin$ or levofloxacin$ or lomefloxacin$ or moxifloxacin$ or nadifloxacin$ or “nalidixic acid” or nemonoxacin$ or  norfloxacin$ or ofloxacin$ or orbifloxacin$ or ozenoxacin$ or “oxolinic acid” or pazufloxacin$ or pefloxacin$ or “pipemidic acid” or “piromidic acid” or pradofloxacin$ or prulifloxacin$ or rosoxacin$ or rufloxacin$ or sitafloxacin$ or sparfloxacin$ or temafloxacin$ or tosufloxacin$ or trovafloxacin$) 4. 2 and 3 5. 1 and 4   Set 5 Limited to Pubyear 2020 & 2021 |
| Web of Science | June 7^th^, 2021 | 1. TS= (campylobacter*) 2. TS= (drug resistance OR resistan*) 3. TS= (fluoroquinolone$ or quinolone$ or besifloxacin$ or cinoxacin$ or ciprofloxacin$ or danofloxacin$ or delafloxacin$ or difloxacin$ or enoxacin$ or enrofloxacin$ or fleroxacin$ or flumequin$ or garenoxacin$ or gatifloxacin$ or gemifloxacin$ or grepafloxacin$ or ibafloxacin$ or levofloxacin$ or lomefloxacin$ or moxifloxacin$ or nadifloxacin$ or “nalidixic acid” or nemonoxacin$ or norfloxacin$ or ofloxacin$ or orbifloxacin$ or ozenoxacin$ or “oxolinic acid” or pazufloxacin$ or pefloxacin$ or “pipemidic acid” or “piromidic acid” or pradofloxacin$ or prulifloxacin$ or rosoxacin$ or rufloxacin$ or sitafloxacin$ or sparfloxacin$ or temafloxacin$ or tosufloxacin$ or trovafloxacin$) 4. 2 and 3 5. 1 and 4 6. Refined 5 to Publication years 2020 & 2021 |
| CAB Abstracts via Ovid | June 7^th^, 2021 | 1. exp campylobacter/ or campylobacter$.ab,hw,ti. 2. exp drug resistance/ or resistan$.ab,hw,ti. 3. exp quinolones/ or (fluoroquinolone$ or quinolone$ or besifloxacin$ or cinoxacin$ or ciprofloxacin$ or danofloxacin$ or delafloxacin$ or difloxacin$ or enoxacin$ or enrofloxacin$ or fleroxacin$ or flumequin$ or garenoxacin$ or gatifloxacin$ or gemifloxacin$ or grepafloxacin$ or ibafloxacin$ or levofloxacin$ or lomefloxacin$ or moxifloxacin$ or nadifloxacin$ or nalidixic acid or nemonoxacin$ or norfloxacin$ or ofloxacin$ or orbifloxacin$ or ozenoxacin$ or oxolinic acid or pazufloxacin$ or pefloxacin$ or pipemidic acid or piromidic acid or pradofloxacin$ or prulifloxacin$ or rosoxacin$ or rufloxacin$ or sitafloxacin$ or sparfloxacin$ or temafloxacin$ or tosufloxacin$ or trovafloxacin$).ab,hw,ti. 4. 2 and 3 5. 1 and 4 6. limit 5 to yr="2020 -Current" |
| Agricola via Ovid | June 7^th^, 2021 | 1. exp campylobacter/ or campylobacter$.ab,hw,ti. 2. exp drug resistance/ or resistan$.ab,hw,ti. 3. exp quinolones/ or (fluoroquinolone$ or quinolone$ or besifloxacin$ or cinoxacin$ or ciprofloxacin$ or danofloxacin$ or delafloxacin$ or difloxacin$ or enoxacin$ or enrofloxacin$ or fleroxacin$ or flumequin$ or garenoxacin$ or gatifloxacin$ or gemifloxacin$ or grepafloxacin$ or ibafloxacin$ or levofloxacin$ or lomefloxacin$ or moxifloxacin$ or nadifloxacin$ or nalidixic acid or nemonoxacin$ or norfloxacin$ or ofloxacin$ or orbifloxacin$ or ozenoxacin$ or oxolinic acid or pazufloxacin$ or pefloxacin$ or pipemidic acid or piromidic acid or pradofloxacin$ or prulifloxacin$ or rosoxacin$ or rufloxacin$ or sitafloxacin$ or sparfloxacin$ or temafloxacin$ or tosufloxacin$ or trovafloxacin$).ab,hw,ti. 4. 2 and 3 5. 1 and 4 6. limit 5 to yr="2020-Current" |
| Global Health via Ovid | June 7^th^, 2021 | 1. exp campylobacter/ or campylobacter$.ab,hw,ti. 2. exp drug resistance/ or resistan$.ab,hw,ti. 3. exp quinolones/ or (fluoroquinolone$ or quinolone$ or besifloxacin$ or cinoxacin$ or ciprofloxacin$ or danofloxacin$ or delafloxacin$ or difloxacin$ or enoxacin$ or enrofloxacin$ or fleroxacin$ or flumequin$ or garenoxacin$ or gatifloxacin$ or gemifloxacin$ or grepafloxacin$ or ibafloxacin$ or levofloxacin$ or lomefloxacin$ or moxifloxacin$ or nadifloxacin$ or nalidixic acid or nemonoxacin$ or norfloxacin$ or ofloxacin$ or orbifloxacin$ or ozenoxacin$ or oxolinic acid or pazufloxacin$ or pefloxacin$ or pipemidic acid or piromidic acid or pradofloxacin$ or prulifloxacin$ or rosoxacin$ or rufloxacin$ or sitafloxacin$ or sparfloxacin$ or temafloxacin$ or tosufloxacin$ or trovafloxacin$).ab,hw,ti. 4. 2 and 3 5. 1 and 4   limit 5 to yr="2020-Current" |
| World Health Organization: Global Index Medicus | June 10^th^, 2021 | (tw:(campylobacter )) AND (tw:(resistan* or antimicrobial resistance or resistance or drug resistance, microbial)) AND (tw:(fluoroquinolone$ or quinolone$ or besifloxacin$ or cinoxacin$ or ciprofloxacin$ or danofloxacin$ or delafloxacin$ or difloxacin$ or enoxacin$ or enrofloxacin$ or fleroxacin$ or flumequin$ or garenoxacin$ or gatifloxacin$ or gemifloxacin$ or grepafloxacin$ or ibafloxacin$ or levofloxacin$ or lomefloxacin$ or moxifloxacin$ or nadifloxacin$ or nalidixic acid or nemonoxacin$ or norfloxacin$ or ofloxacin$ or orbifloxacin$ or ozenoxacin$ or oxolinic acid or pazufloxacin$ or pefloxacin$ or pipemidic acid or piromidic acid or pradofloxacin$ or prulifloxacin$ or rosoxacin$ or rufloxacin$ or sitafloxacin$ or sparfloxacin$ or temafloxacin$ or tosufloxacin$ or trovafloxacin$))   - Year range filter of 2020-2021 was applied |
| Google Scholar | June 10^th^, 2021 | ((campylobacter) AND (("antimicrobial resistance" or resistant* or "drug resistance") AND (quinolone or fluoroquinolone)))   - Year range filter of 2020 to present was applied |

^*^ The same search was run on February 6^th^, 2020 with no date restrictions and a human filter in MEDLINE® via Ovid and EMBASE® via Ovid. In this updated search, MEDLINE® via Ovid and EMBASE® via Ovid were run without the human filter and with a publication date filter from 2018-present. This was done to ensure that all articles were captured even if there was a delay in indexing.

# **Table S2**. Primary screening form at the title/abstract level to identify literature associated with human (fluoro)quinolone-resistant *Campylobacter* spp. infections

| **Logic Chart** | **Question** |
| --- | --- |
| Confirm study population | Is the study population human? □ yes □ no □ unsure |
| Confirm bacteria | Do study participants have *Campylobacter* spp. infections? *□ yes □ no □ unsure* |
| Confirm exposure | Do some or all study participants have (fluoro)quinolone-resistant *Campylobacter* spp. infections? *□ yes □ no □ unsure* |
| Confirm study design | Is the article a primary observational study design?  *□ yes □ no □ unsure* |
| Confirm outcome | Does the study specify one or more direct or indirect burden of illness measures?  *□ yes □ no □ unsure* |
| Confirm language | Is the study published in English or French? *□ yes □ no, specify: ____ □ unsure* |

# **Table S3.** Secondary screening form at the full-text level to identify literature associated with human (fluoro)quinolone-resistant *Campylobacter* spp. infections

| **Logic Chart** | **Question** |
| --- | --- |
| Confirm procurement | Is full-text available?  *□ yes □ no (short conference/abstract only) □ no (manuscript NA)* |
| Confirm language | Is the study published in English or French? *□ yes □ no, specify:______* |
| Confirm study design | Is the article a primary observational study design?  *□ yes □ no* |
| Confirm study population | Is the study population human? □ yes □ no |
| Confirm population/exposure | Do some or all study participants have (fluoro)quinolone-resistant (FQR) *Campylobacter* spp. infections? *□ yes □ no* |
| Confirm comparator | Does the study, when appropriate, include (fluoro)quinolone-susceptible (FQS) *Campylobacter* spp. infections as a comparator? *□ yes □ no (neutral)* |
| Confirm outcome | Does the study specify one or more direct or indirect burden of illness measures related to FQR/FQS cases?  *□ yes □ no* |

# **Table S4.** Data extraction form annotations and an a-priori record of decisions to identify literature associated with human (fluoro)quinolone-resistant *Campylobacter* spp. infections

| **#** | **Study Characteristics** | **Annotations** |
| --- | --- | --- |
| 1. | What is the year of publication? [free text] |  |
| 2. | What year(s) were the data collected? [free text] |  |
| 3. | What is the author reported study design?  [radio: cohort/case-control/cross-sectional/other/not reported] |  |
| 5. | What are the studies objective(s)? [free text] | Author specified, objective statement often located at the end of introduction. |
| 6. | What are the type and number of site(s) included in the study?  [radio: single-site/two-sites/multi-site] | Relates to where cases selected from (e.g., hospital, registry) |
| 7. | What *Campylobacter spp.* were included in the study? [free text] | Enter subspecies (Example: jejuni) |
| 8. | What countries were the study participants enrolled in? [free text] | Capitalize the first letter of the country (Example: Canada). If multiple, list in alphabetic order. |
| # | **Study Participant Characteristics** | **Annotations** |
| 9. | Are there any underlying common disease process and/or common characteristics in the participants with resistant or susceptible infections? [free text] | Are participants of R or S infections of a particular sub-population (i.e., immunocompromised via HIV) |
| 12. | What is the definition of cases with resistant infections? [free text] | Author reported definition |
| 13. | What is the # of cases with resistant infections? | Numeric value |
| 14. | What is the definition of cases with susceptible infections? [free text] | Author reported definition |
| 15. | What is the # of cases with susceptible infections? [free text] | Numeric value |
| 16. | How were cases and comparators selected? [free text] |  |
| 17. | What types of infections are reported in the study?  [radio: gastrointestinal tract infection/blood-stream infection/other, specify] |  |
| 18. | What age details were reported for the case group?  [multi-select with text entry: mean/median/age range/ other, specify] | There is a wide variability in how this data will be reported. |
| 19. | What are the measures of variability reported for cases?  [multi-select with text entry: SD/IQR/NR] |  |
| 20. | What age details were reported for the comparator group?  [multi-select with text entry: mean/median/age range/other, specify]. |  |
| 21. | What are the measures of variability reported for comparators?  [multi-select with text entry: SD/IQR/NR] |  |
| 22. | What age details were reported for participants (combined)? [multi-select with text entry: mean/median/age range/ other, specify] | If exact numbers given for participants in each group, calculate the mean or median. If combine value reported for all participants report here. |
| 23. | What are the measures of variability reported for participants?  [multi-select with text entry: SD/IQR/NR] |  |
| 25. | What were the proportion of females and/or males included in the study? [free text] |  |
| 27. | What is the method used for antimicrobial susceptibility testing? [multi-select: broth micro dilution/disk diffusion/ NR/ Other, specify] |  |
| 28. | What MIC interpretive criteria was used?  [multi-select: CLSI/EUCAST/NR/Other, specify] |  |
| 29. | Specify if quinolone or fluoroquinolone resistant infection(s)  [multi-select: quinolones/fluoroquinolones] |  |
| 30. | What are the self-identified AMR burden study perspectives?  [multi-select: patient/healthcare system/ economic or societal burden/NR/Other, specify] | These categories will most likely not be reported in the study. It will require an assessment of the studies objectives and health impact/outcome factors studied in cases/controls.  Categories adapted from Naylor et al. (2018) paper. Access [Here](https://link.springer.com/article/10.1186/s13756-018-0336-y).  **Patient Perspective:** mortality and morbidity (including clinical outcomes)  **Healthcare System Perspective**: Burden to certain providers of healthcare, such as hospital and primary care practices, length of hospital stay and healthcare costs.  **Economic Perspective**: impact on labour force through lost productivity, burden on carers, patient out of pocket expenses. |
| **#** | **Burden of Illness Results** | **Annotations** |
| 31. | What burden of illness (BOI) measures were reported?  [multi-select: absenteeism/bacteremia/bloody stool/complications/ diarrhea/duration of illness/fever/hospitalization/length of hospital stay/mortality/treatment failure/vomiting/other, specify]  30.1. What definition was used? [free text]  30.2 What were the results? [free text] | The words “burden of illness” will rarely be used to describe these outcome measures.  Selecting one, or multiple, will result in a sub-form for results. If the impact or health outcome measured is not on this list, report in under the “other category”.  30.1. Author reported definition of BOI  30.2. Brief summary of results for the reported BOI. As this paper is not quantifying the results of BOI measures, a summary of the findings will suffice. |
| **#** | **General Comments** | **Annotations** |
| 58. | Please provide any additional information below [free text] |  |

# **Table S5**. Age and sex characteristics of references (excluding case reports) included in scoping review of BOI measures associated with human (fluoro)quinolone-resistant *Campylobacter* spp. infections

| **Reference** | **Age details of case group (R)** | **Measures of Variability** | **Age details of comparator group (S)** | **Measures of Variability** | **Age details of participants (combined)** | **Measures of Variability** | **Proportion (%) of males**  **and/or females included in the study** |
| --- | --- | --- | --- | --- | --- | --- | --- |
| *Campylobacter* Sentinel Surveillance Scheme Collaborators (2002). [1] | Mean- Travel group: 39.0; Indigenous group: 40.1 | NR | Mean- Travel group: 38.0; Indigenous group: 37.9 | NR | NR | NR | Travel group: FQR-47% male, FQS-49% male; Indigenous group: FQR-48% male, FQS-50% male |
| Engberg et al., (2004). [2] | Mean: 31 | IQR: 20-45 | NR | NR | NR | NR | FQR male to female ratio: 1:1.3 |
| Evans et al., (2009). [3] | Median: 53, Age range: 1-86 | NR | Median: 49, Age range: 0-88 | NR | Median: 50, Age range: 0-88 | NR | 51% male |
| Feodoroff et al., (2008). [4] | NR | NR | NR | NR | Median: 39 | NR | 42% male |
| Gaudreau et al., (2013). [5] | Age range: 20-59 | NR | NR | NR | NR | NR | 100% male |
| Gupta et al., (2004). [6] | Median: 46, Age range: 9-76 | NR | Median: 24, Age range: 1-87 | NR | NR | NR | FQR 40% female, FQS 42% female |
| Helms et al., (2005). [7] | NR | NR | NR | NR | NR | NR | NR (demographic data not presented for resistant vs susceptible cases or for combined) |
| Molina et al., (1995). [8] | 30 year old | NR | Mean: 43  Median: 53 | NR | NR | NR | 75% male |
| Mori et al., (2014). [9] | 26 year old, 74 year old, 77 year old | NR | Mean: 45  Median: 54 | NR | Median: 54, Age range: 19-77 | NR | 71% male |
| Nelson et al., (2004). [10] | Mean: 36, Median: 31 | (25th–75th percentile) of the mean: 24-47 | Mean: 34, Median: 34 | (25th–75th percentile) of the mean: 18-49 | Mean: 34, Age range: <1-96 | NR | FQR 51% male FQS 54% male |
| Ricotta et al., (2014). [11] | NR | NR | NR | NR | Mean- No international travel: 36.19; international travel: 37.13,  Age range- No international travel: 0-101.4; International travel: 0-90.1 | NR | No international travel: 55% male; International travel: 53% male |
| Sanders et al., (2002). [12] | NR | NR | NR | NR | Mean: 28.4 | NR | 92% male |
| Smith et al., (1999). [13] | NR | NR | NR | NR | NR | NR | NR |
| Tee & Mijch (1998). [14] | Age range: 28-59 | NR | Age range: 22-77 | NR | NR | NR | HIV cases: 100% male; HIV-negative cases: 67% male |
| Unicomb et al., (2006). [15] | NR | NR | NR | NR | Median: 32 Age range: 0-93 | NR | 52% male |

**IQR:** Interquartile range **FQR:** Fluoroquinolone-resistant

**FQS:** Fluoroquinolone-susceptible

**NR:** Not reported (Information for this field was not provided in the article)

# **Table S6**. Definitions of (fluoro)quinolone resistant infections, method used for antimicrobial susceptibility testing (AST), and AST interpretive criteria identified in all references included in scoping review of BOI measures associated with human (fluoro)quinolone-resistant *Campylobacter* spp. infections

| **Reference** | **Specify if quinolone or fluoroquinolone resistant infection(s)** | **Definition of cases with (Fluoro)quinolone-resistant Infections** | **Method used for antimicrobial susceptibility testing (AST)** | **AST interpretive criteria used** |
| --- | --- | --- | --- | --- |
| *Campylobacter* Sentinel Surveillance Scheme Collaborators (2002). [1] | Fluoroquinolones | Resistant to ciprofloxacin | Agar dilution method | Resistant: agar dilution breakpoints for ciprofloxacin (1mg/L) |
| Engberg et al., (2004). [2] | Quinolones | Resistant to nalidixic acid | Disk diffusion, Other: E-test | NR |
| Evans et al., (2009). [3] | Fluoroquinolones | Resistant to ciprofloxacin | Disk diffusion | British Society for Antimicrobial Chemotherapy guidelines |
| Feodoroff et al., (2008). [4] | Fluoroquinolones | Resistant to ciprofloxacin | Broth microdilution | CLSI clinical breakpoints |
| Gaudreau et al., (2013). [5] | Quinolones, fluoroquinolones | Resistant to ciprofloxacin and nalidixic acid (also resistant to tetracycline and cefotaxime) | Disk diffusion, Other: E-test | CLSI clinical breakpoints |
| Goyal et al., (2021). [16] | Quinolones, fluoroquinolones | Resistant to ciprofloxacin, levofloxacin, nalidixic acid | Broth microdilution | CLSI clinical breakpoints, EUCAST ECOFF |
| Gupta et al., (2004). [6] | Fluoroquinolones | Resistant to ciprofloxacin | E-test | CLSI clinical breakpoints |
| Hartman et al., (2020). [17] | Fluoroquinolones | Two subsequent stool cultures showed resistance to ciprofloxacin | NR | NR |
| Helms et al., (2005). [7] | Quinolones | Resistant to nalidixic acid | Tablet diffusion, (tablet diffusion on Danish blood agar (SSI Diagnostica) by use of  Neosensitabs (Rosco)) | Resistant: inhibition zone of < 27 mm for nalidixic acid |
| Kaneko et al., (2000). [18] | Fluoroquinolones | Resistant to fosfomycin and norfloxacin, but susceptible to ampicillin, erythromycin and gentamicin. | NR | NR |
| Kotilainen et al., (2006). [19] | Fluoroquinolones | Resistant to ciprofloxacin | NR | NR |
| Lau et al., (2002). [20] | Fluoroquinolones | Resistant to ciprofloxacin | NR | NR |
| Magaz-Martinez et al., (2016). [21] | Quinolones | Quinolone-resistant | NR | NR |
| Martora et al., (2020). [22] | Fluoroquinolones | Resistant to ciprofloxacin, doxycycline and tetracycline | Vitek 2 system | NR |
| Molina et al., (1995). [8] | Quinolones | Resistant to quinolones (naladixic acid, norofloxacin, ofloxacin, ciprofloxacin). | Disk diffusion, Other: agar diffusion | NR |
| Mori et al., (2014). [9] | Fluoroquinolones | Susceptibility based on standard agar disk diffusion method | Disk diffusion | NR |
| Nelson et al., (2004). [10] | Fluoroquinolones | Resistant to ciprofloxacin | Other: E-test | Resistant: MIC of 4 µg/mL |
| Nishikubo et al., (2021). [23] | Fluoroquinolones | Resistant to ciprofloxacin | Broth microdilution | NR |
| Dan & Parizade, (2020). [24] | Fluoroquinolones | Resistant to ciprofloxacin, erythromycin, tetracycline and fosfomycin | Other: E-test | CLSI clinical breakpoints |
| Pascual et al., (1994). [25] | Quinolones, fluoroquinolones | Resistant to quinolones and ciprofloxacin | Disk diffusion, Other: E-test | NR |
| Ricotta et al., (2014). [11] | Quinolones, fluoroquinolones | Resistant to ciprofloxacin and nalidixic acid | NR | CLSI clinical breakpoints |
| Sanders et al., (2002). [12] | Fluoroquinolones | Resistant to ciprofloxacin | NR | NR |
| Smith et al., (1999). [13] | Quinolones, fluoroquinolones | Resistant to ciprofloxacin and nalidixic acid | Disk diffusion, Other: E-test | CLSI clinical breakpoints |
| Tajada et al., (1997). [26] | Fluoroquinolones | Resistant to ciprofloxacin | Other: Agar dilution | NR |
| Tee & Mijch (1998). [14] | Fluoroquinolones | Resistant to ciprofloxacin | Disk diffusion | NR |
| Unicomb et al., (2006). [15] | Fluoroquinolones | Resistant to ciprofloxacin | Other: Agar dilution | CLSI clinical breakpoints |

**CLSI:** Clinical and Laboratory Standards Institute
**EUCAST**: European Committee on Antimicrobial Susceptibility Testing
**ECOFF:** Epidemiological Cut-Off Value
**MIC:** Minimum Inhibitory Concentration

# **Table S7**. Identified comparisons of BOI measures and other findings in human (fluoro)quinolone-resistant and susceptible *Campylobacter* spp. infections

| **BOI Perspective** | **BOI measures category** | **References where BOI is greater among resistant infections** | **References where BOI is greater among susceptible infections** | **References where there is no difference between resistant and susceptible infections/other findings** | **References with other findings in resistant infections** |
| --- | --- | --- | --- | --- | --- |
| Patient | Diarrhea | - | - | No difference: CIP-R and CIP-S (94.5% vs 95.4% P=0.84) [3] | CIP-R *C. jejuni* and *C. coli* isolates were not associated with severe disease, i.e., disease with diarrhea lasting for >10 days [4] |
|  |  |  |  | HIV+ (2/2 FQR and 6/7 FQS) and HIV- (1/1 FQR and 9/11 FQS) [14] |  |
|  | Fever | - | CIP-R less likely to report fever (OR, 0.5; 95% CI, 0.3-0.7; P<0.001) than CIP-S, and the reduced likelihood of fever in CIP-R (aOR, 0.5; 95% CI, 0.3-0.8) remained after adjustment for age, sex, and foreign travel [3] | HIV+ (2/2 FQR and 7/7 FQS) and HIV- (1/1 FQR and 9/11 FQS) [14] | - |
|  |  |  |  | No difference: CIP-R and CIP-S (68% vs 74%; OR, 1.6; 95% CI, 0.4-5.9) [15] |  |
|  |  |  |  | No difference: CIP-R (63/76, 83%) and CIP-S (526/630, 83%) (P=0.87) [10] |  |
|  | Duration of illness | Mean duration of diarrhea: CIP-R (8 days (range, 2–21 days; median, 7 days; IQR, 5-10)) and CIP-S (7 days (range, 1–60 days; median, 6 days; IQR 4-8, P=0.10), and multivariable analysis (adjustment for antimicrobial medication, antidiarrheal medication, and antacid use): CIP-R and CIP-S (9 vs 8 days, P=.01) [10] | - | No difference in mean duration of diarrhea: CIP-R (8.2 days (median, 6.0 days; range, 2–35 days)) and CIP-S (8.6 days (median, 7.0 days; range, 1–42 days), P=0.57), and >7-day duration of diarrhea in CIP-R and CIP-S (48% vs 52%, P=0.43) [3] | - |
|  |  | Median duration of diarrhea: Quin-R and Quin-S *C. jejuni* infections (10 vs 7 days, P=0.03) [13]* |  | No difference in median duration of diarrhea: both CIP-R and CIP-S (7 days, P=0.63) in a multivariate model adjusted for age and underlying disease, regardless of travel status [15] |  |
|  |  | Median duration of illness: significantly longer for Quin-R *C. jejuni* infections than for Quin-S infections (13.2 vs 10.3 days, p=0.001) [2]* |  | No difference in mean duration of illness Quin-R and Quin-S *C. coli* infections [2] |  |
|  |  |  |  | No difference in mean length of illness between CIP-R and S in travel-associated *C. jejuni* cases (12.7 versus 13.5 days; *t*-test P = 0.56) and between CIP-R and S Indigenous *C. jejuni* cases (11.8 versus 11.2 days; *t*-test P = 0.66) [1] |  |
|  |  |  |  | Median duration of diarrhea:  HIV+ (5 days to ongoing FQR and 1 day to ongoing FQS) and HIV- (3 days FQR and 1->30 days FQS) [14] |  |
|  |  |  |  | Median duration of fever:  HIV+ (3 days to ongoing FQR and 1-3 days FQS) and HIV- (3 days FQR and 1-28 days FQS) [14] |  |
|  | Bloody stool | Bloody diarrhea: CIP-R (8/14, 57%) and CIP-S (8/27,30%) (mOR=3.2, 95% CI, 0.8-12.1) [6] | CIP-S isolates seemed to associate with a more invasive disease, as characterized by bloody stools (67%) [4] | No difference between CIP-R and CIP-S (22.0% vs 25.3% P=0.50) [3] | CIP-R *C. jejuni* and *C. coli* isolates were not associated with severe disease, i.e. disease with bloody stools [4] |
|  |  |  |  | No difference between CIP-R and CIP-S (15% vs 42%; OR, 0.4; 95% CI, 0.1-5.4) [15] |  |
|  |  |  |  | No difference between CIP-R (35/77, 45%) and CIP-S (264/612, 43% P=0.72) [10] |  |
|  | Abdominal pain | - | - | No difference between CIP-R and CIP-S (86.9% vs 88.6%, P=0.70) [3] | - |
|  |  |  |  | No difference between CIP-R and CIP-S (68/78, 87% vs 544/640, 85%, P=0.74) [10] |  |
|  | Treatment failure | CIP-R initial treatment CIP: 11/19 (58%) cured, 5/19 (26%) not cured at 72 hours and changed to azithromycin, and 3/19 (16%) lost to follow-up. CIP-S initial treatment CIP: 1/1 (100%) cured [12] | - | - | Diarrhea continued through treatment with ciprofloxacin, and several other antibiotics, but resolved with 7 days of imipenem/cilastatin [16] |
|  |  |  |  |  | CIP-R lead to other treatments including clarithromycin in (patient 1) and amoxicillin/clavulanic acid combined with sulfamethoxazole/trimethoprim (patient 2) [22] |
|  |  |  |  |  | Patient with diarrhea received multiple 10-14 day courses of ciprofloxacin and azithromycin, but remained symptomatic and culture positive for *C. coli* until cured with gentamicin [24] |
|  |  |  |  |  | Patient with diarrhea, bacteremia and femoral pain not cured with ciprofloxacin and other antibiotics, and resolved with meropenem [17] |
|  | Vomiting | - | - | No difference between CIP-R and CIP-S (26.9% vs 32.1%, P=0.29) [3] | - |
|  |  |  |  | No difference between CIP-R and CIP-S (24/79, 30% vs 182/648, 28%, P=0.69) [15] |  |
|  |  |  |  | No difference between CIP-R and CIP-S (38% vs 35%; OR, 1.6; 95% CI, 0.6-4.5) [10] |  |
|  | Complications | - | - | - | Stool culture positive for *C. jejuni* and developed diabetic ketoacidosis after failed treatment with ciprofloxacin [16] |
|  |  |  |  |  | Osteomyelitis due to FQR *C. jejuni* [17] |
|  |  |  |  |  | Myopericarditis in association with *Campylobacter* spp. enteritis that was CIP-R [19] |
|  |  |  |  |  | Vertebral osteomyelitis and breast implant infection due to *C. fetus* that developed Quin-R during treatment [23] |
|  | Mortality | - | - | HIV+ (2/2 FQR and 2/7 FQS) and HIV- (0/1 FQR and 9/11 FQS) [14] | Patient died in hospital and isolation of Quin-R *Campylobacter jejuni* was confirmed after death [48] |
|  |  |  |  | Quin-R 1/1 and Quin-S 1/3 [8] |  |
|  | Nausea | - | - | No difference between CIP-R and CIP-S (51.0% vs 58.2%, P=0.17) [3] | - |
|  | Other: |  |  |  |  |
|  | Muscle aches | - | - | No difference between CIP-R and CIP-S (49.0% vs 49.9% P=0.93) [3] | - |
|  | Painful joints | - | - | No difference between CIP-R and CIP-S (41.4% vs 40.6% P=0.95) [3] |  |
|  | Weakness | - | - | No difference between CIP-R and CIP-S (79.3% vs 81.3% P=0.70) [3] |  |
|  | Headache | - | - | No difference between CIP-R and CIP-S (47.6% vs 52.1% P=0.41) [3] |  |
|  | Adverse events (invasive illness or death) | Within 30 days of infection, patients with Quin-R *Campylobacter* spp. infection had a 6.17-fold (95% CI, 1.62–23.47) increased risk of adverse events (diagnosis of either invasive illness or death), after adjustment for sex, age, and comorbidity, compared with patients infected with quinolone- and erythromycin-susceptible *Campylobacter* spp. strains, and patients infected with a Quin-R *Campylobacter* spp. strain had a 3-fold (95% CI, 0.99–9.39) increased risk of adverse events within 90 days of infection, after adjustment for sex, age, and comorbidity, compared with patients infected with quinolone-and erythromycin-susceptible *Campylobacter* spp. strains [7] | - | - | - |
|  | Reduced appetite, Night sweats, Severe polar | - | - | - | CIP-R *C. jejuni* in two patients where one case experienced bouts of reduced appetite and the other case presented with night sweats and severe polar [22] |
|  | Progressive pain in upper extremities due to osteomyelitis | - | - | - | Progressive pain in both upper extremities and diagnosed FQR *C. jejuni* osteomyelitis [17] |
|  | Ileocolitis | - | - | - | Fatal case of infectious active ileocolitis with isolation of Quin-R *C. jejuni* [21] |
|  | Pharyngotonsillitis | - | - | - | Pharyngotonsillitis with isolation of FQR *C. jejuni* [26] |
|  | Urinary tract infection | - | - | - | UTI where Quin-R *C. coli* was cultured from urine [25] |
| Healthcare System | Hospitalization | CIP-R (5/16 [31%]), *C. jejuni* infections were more likely to be hospitalized compared to CIP-S (1/31 [3%]), [mOR]=13.6, 95% CI, 1.4-130.1) based on a small number of patients, with age a possible confounder [6] | CIP-S isolates seemed to associate with a more invasive disease which was reflected in a higher number of patients reporting hospitalization [4] | No difference between CIP-R and CIP-S (9.0% vs 8.5% of patients, P>0.99) [3] | CIP-R *C. jejuni* and *C. coli* isolates were not associated with severe disease, i.e. leading to hospital treatment [4] |
|  |  |  | International travel-associated Quin-R *Campylobacter* spp. isolates (5.8%) whereas Quin-S *Campylobacter* spp. was higher (6.8%) [11] | No difference between CIP-R and CIP-S (14% versus 13%; OR, 0.8; 95% CI, 0.2-3.6) [15] |  |
|  |  | Among non-travel associated cases, these proportions were 20% for Quin-R *Campylobacter* spp. isolates, and 18% for Quin-S *Campylobacter* spp. isolates [11] |  | No difference between CIP-R and CIP-S (10/82, 12% vs 82/654, 12%, P=1.0) [10] | In a multivariable analysis with travel as an additional covariate, they found that antibiotic resistance did not appear to be significantly associated with odds of requiring hospitalization (ciprofloxacin OR, 0.96; 95% CI, 0.75-1.21; P=0.728) [11] |
|  |  |  |  | No difference in admission to hospital between cases with CIP-R and CIP-S in travel-associated *C. jejuni* infections (both 6%; χ^2^ P = 0.9) and Indigenous *C. jejuni* infections (14% versus 12%; χ^2^ P = 0.39) [1] |  |
|  | Length of hospital stay | - | Mean LOS was longer for CIP-S (3 days, IQR=2-4) than CIP-R (2 days, IQR=1-3, P=0.01) [10] | Median LOS: 0 days for both CIP-R and CIP-S (P=0.13) in a multivariate model controlling for age and underlying disease, regardless of travel status [15] | Multivariable analysis adjusted for international travel and found that CIP-R was not significantly associated with LOS (OR=1.07; 95% CI=0.90, 1.29; P=0.427) [11] |
| Economic | Absenteeism | - | - | Mean duration of time off work or school: no difference between CIP-R and CIP-S (8.3; median,7 vs 8.6; days, median, 6; P=0.84) [30]  Missed work or school: no difference between CIP-R and CIP-S (57% vs 60.7%, P=0.58) [3] | - |
|  |  |  |  | Duration of days of missed work: no difference between CIP-R and CIP-S (mean of 3 days, IQR=2-4 vs of 4 days, IQR=2-5, P=0.40 and a median of 3 days for both (P=0.20) [34]  Duration of days of missed usual activities: no difference between CIP-R and CIP-S (mean 6 days in both, IQR=3-6 vs IQR=3-7, P=0.80) and median 4 days vs 5 days, P=0.30) [10] |  |

**aOR:** Adjusted odds ratio

**CIP-R:** Ciprofloxacin-resistant

**CIP-S:** Ciprofloxacin-susceptible

**FQR:** Fluoroquinolone-resistant

**FQS:** Fluoroquinolone-susceptible

**LOS:** Length of stay

**mOR:** Matched odds ratio

**Quin-R:** Quinolone-resistant

**Quin-S:** Quinolone-sensitive

**S**: Susceptible

-None

*Reported as statistically significant

**Table S8**. Key information to collect when assessing BOI from (fluoro)quinolone-resistant and susceptible *Campylobacter* spp. infections in humans

| **Key information** | **Details** |
| --- | --- |
| Infection | - Sample source - Species - Method used for antimicrobial susceptibility testing (AST) - AST interpretive criteria |
| Patient Characteristics and confounders | - Age - Sex - Co-morbidities/Underlying disease |
| BOI measures with definitions and stratified by resistant and susceptible cases | - Mortality - Morbidity - Healthcare system - Economic impact |

References

1. ***Campylobacter* Sentinel Surveillance Scheme Collaborators**. (2002) Ciprofloxacin resistance in *Campylobacter jejuni*: case-case analysis as a tool for elucidating risks at home and abroad. *Journal of Antimicrobial Chemotherapy*; **50**: 561-568. doi:10.1093/jac/dkf173.
2. **Engberg J, *et al****.* (2004) Quinolone-resistant *Campylobacter* infections in Denmark: risk factors and clinical consequences. *Emerging Infectious Diseases*; **10**: 1056-1063. doi:10.3201/eid1006.030669.
3. **Evans MR, *et al***. (2009) Short-term and medium-term clinical outcomes of quinolone-resistant *Campylobacter* infection. *Clinical Infectious Diseases*; **48**: 1500-1506. doi:10.1086/598932.
4. **Feodoroff FBL, *et al****.* (2008) Severe diarrhoea caused by highly ciprofloxacin-susceptible *Campylobacter* isolates. *Clinical Microbiology and Infection*; **15**: 188-192. doi:10.1111/j.1469-0691.2008.02657.x.
5. **Gaudreau C, *et al.*** (2013) *Campylobacter coli* outbreak in men who have sex with men, Quebec, Canada, 2010-2011. *Emerging Infectious Diseases*; **19**: 764-767. doi: 10.3201/eid1905.121344.
6. **Gupta A, *et al*.** (2004) Antimicrobial resistance among *Campylobacter* strains, United States, 1997-2001. *Emerging Infectious Diseases*; **10**: 1102-1109. doi:10.3201/eid1006.030635.
7. **Helms M, *et al.*** (2005) Adverse health events associated with antimicrobial drug resistance in *Campylobacter* species: a registry-based cohort study. *The Journal of Infectious Diseases*; **191**: 1050-1055. doi:10.1086/428453.
8. **Molina JM, *et al.*** (1995) *Campylobacter* infections in HIV-infected patients: clinical and bacteriological features. *AIDS*; **9**: 881-885. doi:10.1097/00002030-199508000-00008
9. **Mori T, *et al.*** (2014) Clinical features of bacteremia due to *Campylobacter jejuni*. *Internal Medicine*; **53**: 1941-1944. doi:10.2169/internalmedicine.53.2559
10. **Nelson JM, *et al.*** (2004) Prolonged diarrhea due to ciprofloxacin-resistant *Campylobacter* infection. *The Journal of Infectious Diseases*; **190**: 1150-1157. doi:10.1086/423282.
11. **Ricotta EE, *et al****.* (2014) Epidemiology and antimicrobial resistance of international travel-associated *Campylobacter* infections in the United States, 2005-2011. *American Journal of Public Health*; **104**: e108-e114. doi:10.2105/AJPH.2013.301867
12. **Sanders *et al.*** (2002) An observational clinic-based study of diarrheal illness in deployed United States military personnel in Thailand: presentation and outcome of *Campylobacter* infection. *The American Journal of Tropical Medicine and Hygiene*; **67**: 533-538. doi:10.4269/ajtmh.2002.67.533.
13. **Smith KE, *et al****.* (1999) Quinolone-resistant *Campylobacter jejuni* infections in Minnesota, 1992-1998. *The New England Journal of Medicine*; **340**: 1525-1531. doi:10.1056/NEJM199905203402001.
14. **Tee W, Mijch A.** (1998) *Campylobacter jejuni* bacteremia in human immunodeficiency virus (HIV)-infected and non-HIV-infected patients: comparison of clinical features and review. *Clinical Infectious Diseases*; **26**: 91-96. doi:10.1086/516263.
15. **Unicomb LE, *et al*.** (2006) Low-level fluoroquinolone resistance among *Campylobacter jejuni* isolates in Australia. *Clinical Infectious Diseases*; **42**: 1368-1374. doi:10.1086/503426.
16. **Goyal D, *et al.*** (2021) Antimicrobial susceptibility testing and successful treatment of hospitalised patients with extensively drug-resistant *Campylobacter jejuni* infections linked to a pet store puppy outbreak. *Journal of Global Antimicrobial Resistance*; **26**: 84-90. doi: 10.1016/j.jgar.2021.04.029.
17. **Hartman J, Westerman M, Wagenaar JFP***.* (2020) Two-sided femoral *Campylobacter jejuni* oseteomyelitis in a patient with acquired hypogammaglobulinemia: a case report. *BMC Infectious Diseases;* **20**: 299. doi:10.1186/s12879-020-4929-8.
18. **Kaneko M, *et al.*** (2000) *Campylobacter jejuni* bacteremia in an immunocompetent Japanese child. *Pediatrics International*; **42**: 579-581. doi:10.1046/j.1442-200x.2000.01280.x.
19. **Kotilainen P, Lehtopolku M, Hakanen, AJ.** (2006) Myopericarditis in a patient with *Campylobacter* enteritis: a case report and literature review. *Scandinavian Journal of Infectious Diseases*; **38**: 549-552. doi: 10.1080/00365540500372903.
20. **Lau SK, *et al****.* (2002) Emergence of cotrimoxazole- and quinolone-resistant *Campylobacter* infections in bone marrow transplant recipients. *European Journal of Clinical Microbiology and Infectious Disease;* **21**: 127-129. doi:10.1007/s10096-001-0673-1.
21. **Magaz-Martinez, *et al.*** (2016). Fatal *Campylobacter jejuni* ileocolitis. *Revista Espanola de Enfermedades Digestivas;* **108**: 662-663.
22. **Martora, *et al.*** (2020). *Campylobacter jejuni* bacteremia in Italian pediatric patients with acute lymphoblastic leukemia: Report of two cases. *New Microbiologica*; **43**: 96-98.
23. **Nishikubo *et al.*** (2021) Sequential breast implant infections due to *Campylobacter fetus* subsp. *fetus*. *Journal of Infection and Chemotherapy*; **27**: 1080-1083. doi:10.1016/j.jiac.2021.01.012
24. **Dan M, Parizade M.** (2020) Chronic high-level multidrug-resistant *Campylobacter coli* enterocolitis in an agammaglobulinemia patient: oral gentamicin efficacy. *Medecine et Maladies Infectieuses;* **50**: 525-527. doi:10.1016/j.medmal.2020.04.013.
25. **Pascual *et al*.** (1994) Urinary tract infection caused by quinolone-resistant *Campylobacter coli.* *European Journal of Clinical Microbiology and Infectious Diseases*; **13**: 690-691.
26. **Tajada P, Alos JI, Gomez-Garces JL.** (1997) Bacteremia due to fluoroquinolone-resistant *Campylobacter jejuni* in an immunocompetent child. *Clinical Microbiology Newsletter*; **19**: 52-53.
